# Manipulation of the precursor supply for high-level production of longifolene by metabolically engineered *Escherichia coli*

**DOI:** 10.1038/s41598-018-36495-w

**Published:** 2019-01-14

**Authors:** Yujin Cao, Rubing Zhang, Wei Liu, Guang Zhao, Wei Niu, Jiantao Guo, Mo Xian, Huizhou Liu

**Affiliations:** 1 CAS Key Laboratory of Biobased Materials, Qingdao Institute of Bioenergy and Bioprocess Technology, Chinese Academy of Sciences, Qingdao, China; 2 Department of Chemistry, University of Nebraska-Lincoln, Lincoln, Nebraska United States

## Abstract

Longifolene is a naturally occurring tricyclic sesquiterpene widely used in many different fields. Up to now, this valuable terpene was mainly manufactured from the high-boiling fraction of certain pine resins. Microbial production can be a promising alternative to the extraction from natural plant sources. Here, we present the metabolic engineering strategy to assemble biosynthetic pathway for longifolene production in *Escherichia coli*. *E. coli* was rendered to produce longifolene by heterologously expressing a codon optimized longifolene synthase from *Picea abies*. Augmentation of the metabolic flux to farnesyl pyrophosphate (FPP) by different FPP synthases conferred a 1.8-fold increase in longifolene production. An additional enhancement of longifolene production (up to 2.64 mg/L) was achieved by introducing an exogenous mevalonate pathway. Under fed-batch conditions, the best-performing strain was able to produce 382 mg/L of longifolene in a 5 L bioreactor. These results demonstrated the feasibility of producing longifolene by microbial fermentation and could serve as the basis for the construction of more robust strains in the future.

## Introduction

Longifolene (C_15_H_24_, decahydro-4,8,8-trimethyl-9-methylene-1-4-methanoazulene), a naturally occurring tricyclic sesquiterpene, has extensive applications in many different fields. Longifolene itself is used in the perfumery industry owing to its special woody fragrances^1^. Longifolene can also serve as an additive for lubricating oils^2^ and insect repellants^3^. It is an important component of the essential oil of *Nigella sativa* which shows antimicrobial activities^4^. Finally, maybe the most important of all, longifolene is a versatile raw material in organic synthesis for the preparation of many perfumery products such as isolongifolene^5^, isolongifolol^6^ and dilongifolylborane^7^. Due to its compact structure, longifolene possess a high density of 0.928 and a high combustion heat. It has the potential to act as a feedstock for advanced biofuels with excellent properties^8^.

The total synthesis of longifolene was accomplished by organic chemists^9,10^ decades ago. However, the intricate carbon framework of this sesquiterpene makes it difficult to be produced by chemical processes. Longifolene is currently manufactured from the high-boiling fraction of certain pine resins^11^. The extraction procedure of longifolene from resins is complex and inefficient, as well as requiring substantial expenditure of natural resources due to its low content. The limitation of raw materials and high energy cost for separation make this process economically unfeasible. Therefore, there is growing interest in developing new routes which could use microorganisms to convert renewable resources into longifolene. Compared with traditional resin extraction methods, microbial synthesis of longifolene offers many technical and economical advantages, e.g., easiness to be cultivated, convenience to be modified via genetic engineering and no requirement for arable land to be planted.

**Figure 1:**
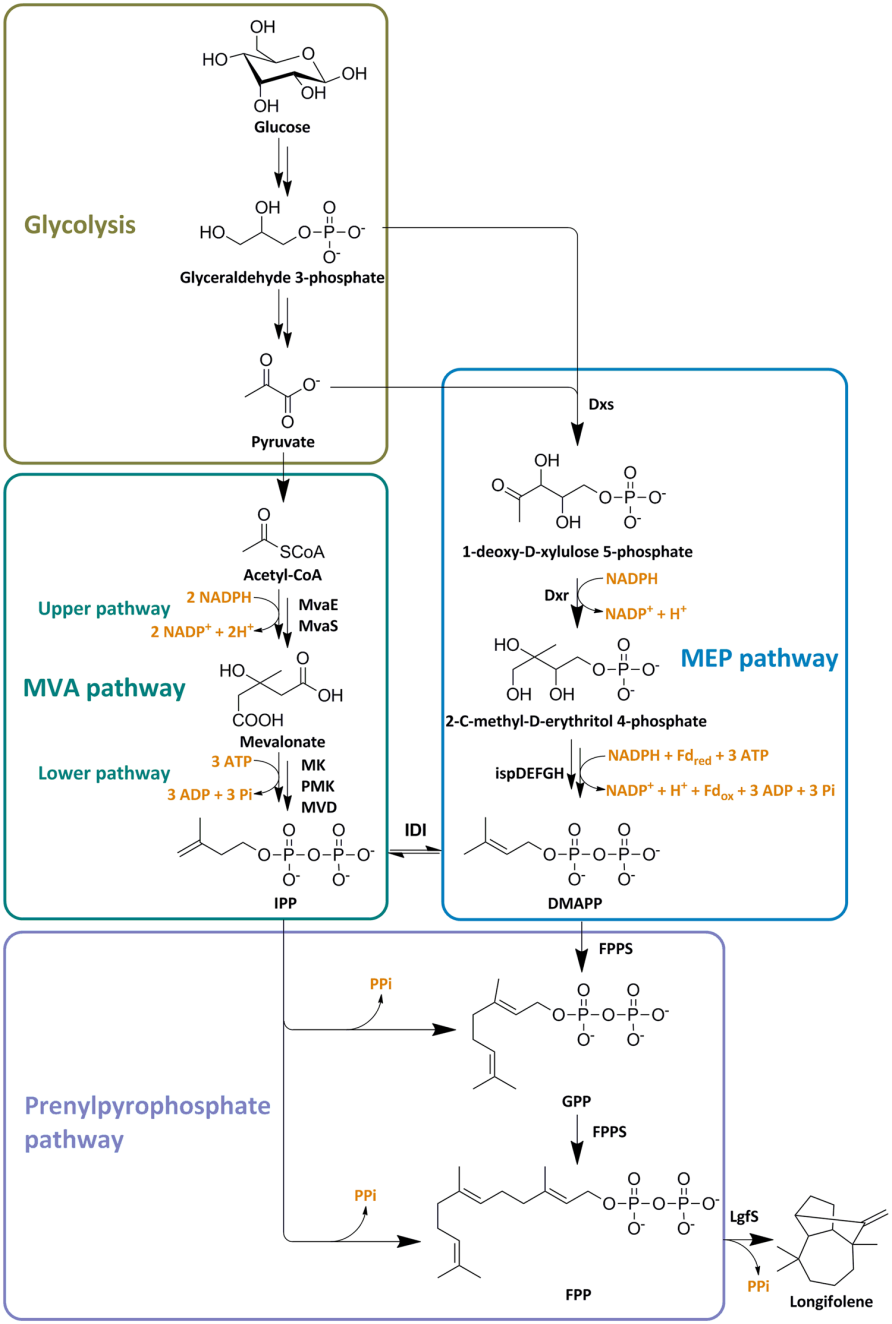
Biosynthetic pathway of longifolene in this study. Enzymes involved in the MEP pathway includes: Dxs, 1-deoxy-D-xylulose-5-phosphate synthase; Dxr, 1-deoxy-D-xylulose 5-phosphate reductoisomerase; IspD, 4-pyrophosphocytidyl-2-C-methyl-D-erythritol synthase; IspE, 4-pyrophosphocytidyl-2-C-methylerythritol kinase; IspF, 2-C-methyl-D-erythritol 2,4-cyclopyrophosphate synthase; IspG, 4-hydroxy-3-methylbut-2-enyl pyrophosphate synthase; IspH, 1-hydroxy-2-methyl-butenyl 4-pyrophosphate reductase. Enzymes involved in the MVA pathway includes: MvaE, acetyl-CoA acetyltransferase/HMG-CoA reductase; MvaS, HMG-CoA synthase; MK, mevalonate kinase; PMK, phosphomevalonate kinase; MVD, mevalonate pyrophosphate decarboxylase; IDI, IPP isomerase; FPPS, farnesyl pyrophosphate synthase; LgfS, longifolene synthase. Reactions responsible for NADPH, ferredoxin (Fd) and ATP consumption are indicated.

Although many plant sesquiterpene synthases have been identified and characterized, few studies have been conducted to produce these compounds by microbial transformation. Up to now, only α-farnesene^12^, β-caryophyllene^13^, bisabolene^14^, valencene^15^ and β-sesquiphellandrene^16^ have been reported to be synthesized using engineered microorganisms. Like other sesquiterpenes, longifolene biosynthesis begins with farnesyl pyrophosphate (FPP) catalyzed by longifolene synthase (LgfS) featuring a cationic polycyclization-dependent reaction mechanism. FPP is generated from the head-to-tail condensation of the common five-carbon precursor, isopentenyl pyrophosphate (IPP) with its isomer dimethylallyl pyrophosphate (DMAPP). In turn, IPP and DMAPP can be produced from the methylerythritol 4-phosphate (MEP) pathway or the mevalonate (MVA) pathway (Fig. 1)^17^. *Escherichia coli* utilizes a native MEP pathway to synthesize IPP and DMAPP. The MEP pathway has a higher theoretical yield, but it is tightly regulated by the hosts. It has been demonstrated that a heterologous MVA pathway was more effective to increase the precursor supply for the production of terpenes in *E. coli*^18^.

The present study was designed to engineer *E. coli* to produce longifolene by heterologously expressing an LgfS from *Picea abies*. A series of genetic modifications were employed, including codon optimization of LgfS to increase its expression level; overexpression of different FPP synthases to enhance the supply of the precursor; and further introduction of a hybrid MVA pathway to augment the pools of IPP and DMAPP, which lead to increases in longifolene production. Finally, the best-performing strain was cultured under fed-batch conditions to evaluate its ability for large-scale production.

## Results

### Detection of longifolene production by introducing a heterologous longifolene synthase

**Figure 2:**
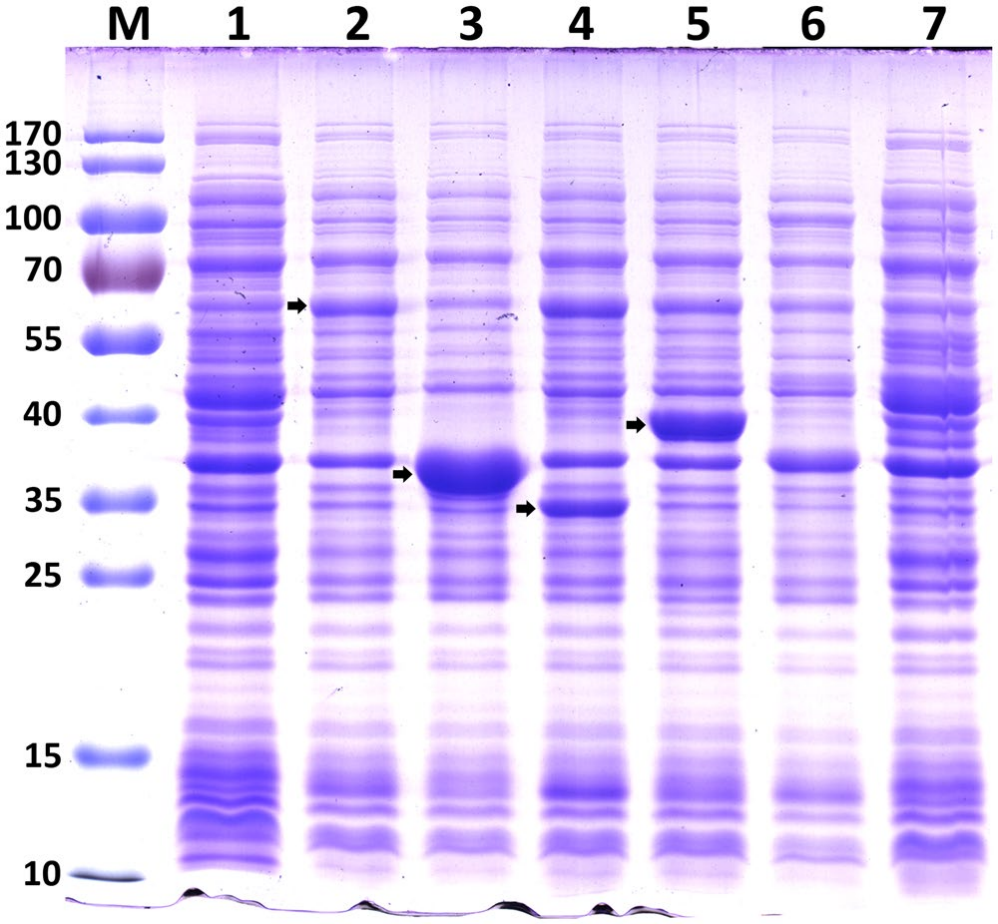
SDS-PAGE analysis of different recombinant proteins for longifolene biosynthesis expressed in *E*. *coli*. Recombinant protein expression was induced using 0.1 mM IPTG for a cultivation time of 4 h at 37 °C. Lane M, protein molecular weight marker; lane 1, control strain harboring pACYCDuet-1; lane 2, crude cells extracts from strain BL21/pA-lgfS; lane 3, crude cells extracts from strain BL21/pA-ispAlgfS; lane 4, crude cells extracts from strain BL21/pA-isoAlgfS; lane 5, crude cells extracts from strain BL21/pA-erg20lgfS; lane 6, crude cells extracts from recombinant strain harboring pTrcLower; lane 7, crude cells extracts from recombinant strain harboring both BL21/pA-mvaESispAlgfS and pTrcLower. The bands corresponding to the individual proteins were indicated by an arrow.

**Figure 3:**
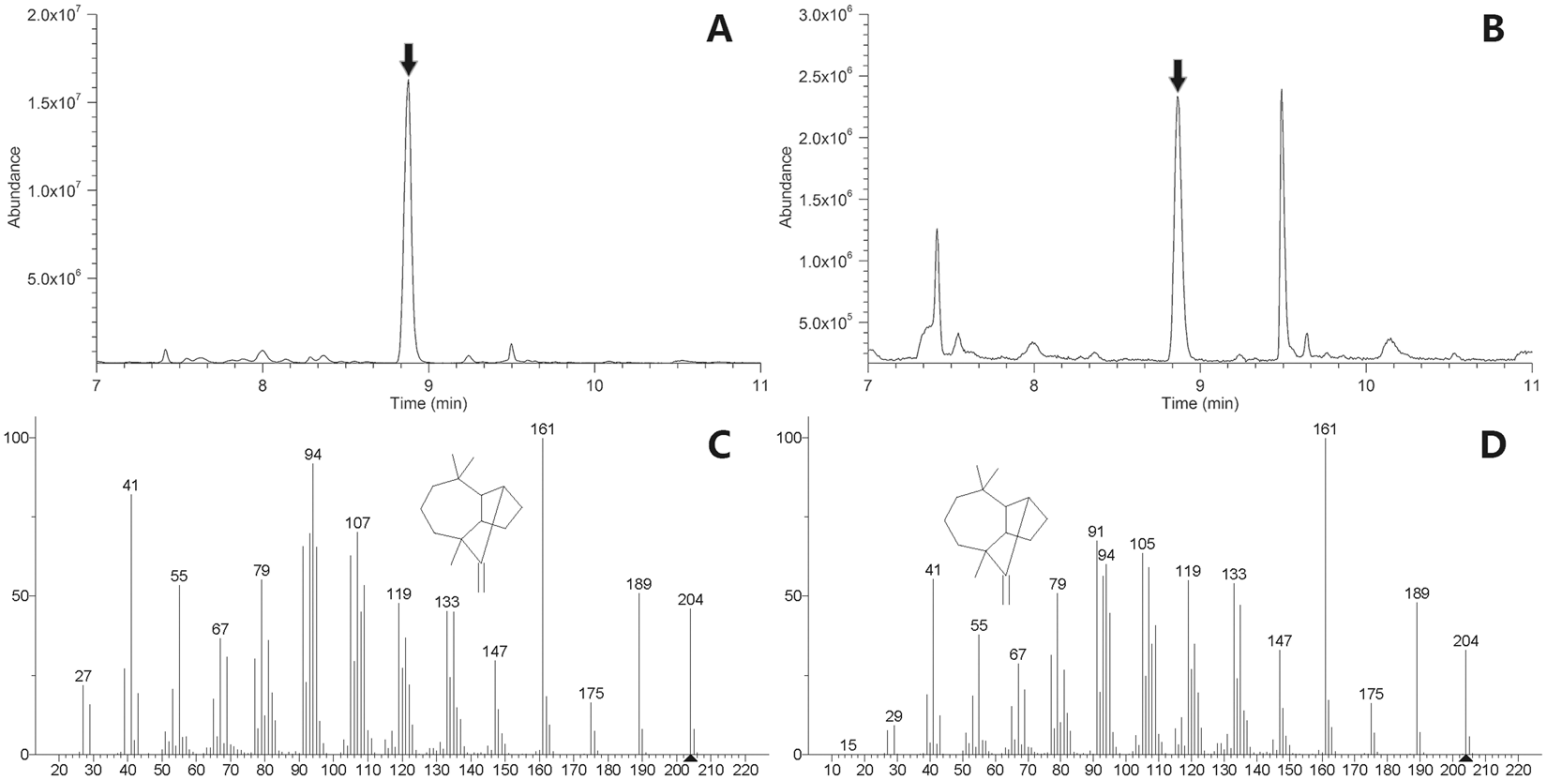
Identification of longifolene by GC-MS. (**A**) total ion current chromatogram of longifolene standard (the peak of longifolene was marked with an arrow corresponding to the retention time of 8.87 min). (**B**) total ion current chromatogram of the gas in the sealed shake flask from *E. coli* BL21(DE3) harboring pA-lgfS after being induced for 24 h. (**C**) mass spectrum of longifolene standard. (**D**) mass spectrum of the fermentation product. The structure of longifolene was matched by searching the NIST 08 library. The molecular ion peak of longifolene was marked with a triangle.

LgfS catalyzes the biosynthesis of longifolene using FPP as the direct substrate through a polycyclization reaction. *E. coli* could maintain a small FPP pool for the synthesis of octaprenyl pyrophosphate (OPP) and undecaprenyl pyrophosphate (UPP) as part of its primary metabolism. OPP is responsible for the synthesis of the side chain of isoprenoid quinones, ubiquinone-8 and demethylmenaquinone-8, which are essential for the respiratory chain^19^. UPP is an important component involved in construction of the peptidoglycan cell wall^20^. LgfS is not expressed by *E. coli* endogenously, but found in plants. Here, we cloned the *lgfS* gene from *P. abies* into the prokaryotic expression vectors, resulting in recombinant plasmids pET-lgfS or pA-lgfS. Because plant terpenoid synthases were always poorly expressed in *E. coli*^21^, the DNA sequence of *lgfS* was codon optimized according to *E. coli*’s preference (Supplementary Figure S1). SDS-PAGE analysis showed that this enzyme was successfully expressed in a soluble form (Fig. 2, lane 2). Then strain BL21/pET-lgfS and BL21/pA-lgfS were tested for longifolene production. As longifolene is a volatile hydrocarbon, fermentation was performed in sealed shake flasks. The culture broth was extracted with hexane and then the organic phase was analyzed by gas chromatography-mass spectrometry (GC-MS). Based on the relative retention time and mass spectra compared with an external standard, trace amount of longifolene was identified (Fig. 3). Two-phase fermentation has been successfully employed to collect water-immiscible or volatile compounds. Therefore, we used hexane as the extraction solvent for two-phase fermentation. The hexane phase of the two-phase culture was collected and centrifuged to remove cell debris, and subsequently subjected to GC-MS. Trace amount of longifolene was also detected. To test longifolene production in the gas phase, the headspace gas of the sealed cultures was measured by gas chromatography (GC) or GC-MS. Much higher level of longifolene (0.90 mg/L) was determined. Although longifolene possesses a high boiling point of 254 °C (706 mm Hg), the vapor pressure of this compact olefin is relatively high^22^. Therefore, most of the longifolene generated by the engineered strain existed in a gas form in the sealed shake flasks. In the following experiments, longifolene was determined in by analyzing the headspace gas or the off-gas.

### Effects of different FPP Synthases on longifolene production

**Figure 4:**
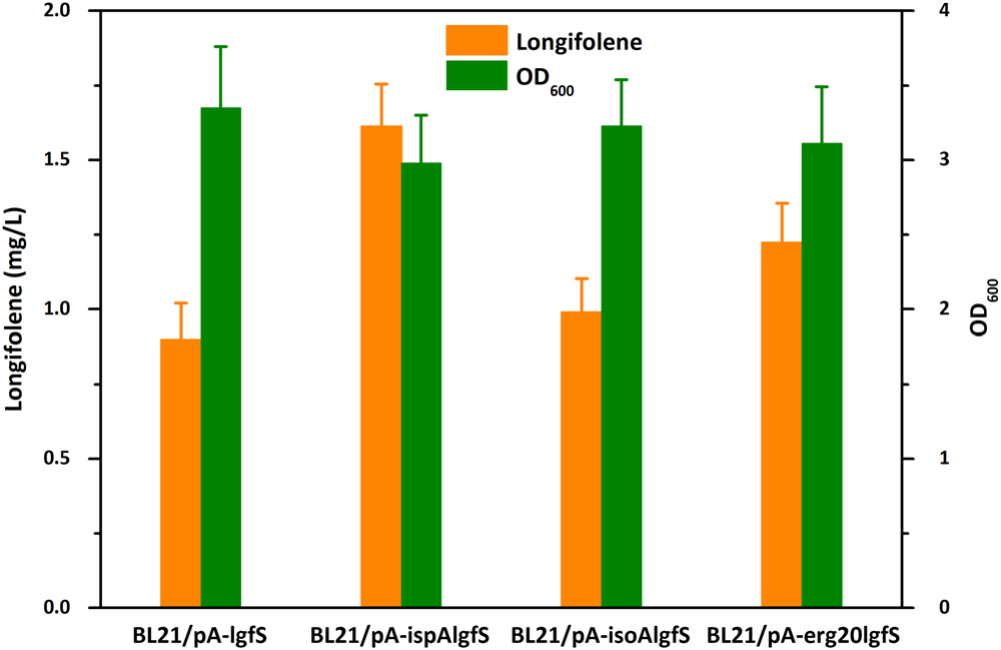
Effects of different FPP synthases on longifolene production. BL21/pA-lgfS, strain BL21(DE3) expressing longifolene synthase; BL21/pA-ispAlgfS, strain BL21(DE3) expressing longifolene synthase and native *E. coli* FPP synthase (IspA); BL21/pA-isoAlgfS, strain BL21(DE3) expressing longifolene synthase and *B. trispora* FPP synthase (IsoA); BL21/pA-erg20lgfS, strain BL21(DE3) expressing longifolene synthase and *S. cerevisiae* FPP synthase (Erg20). Data were obtained after each strain was induced for 24 h in liquid M9 mineral medium supplemented with 1 mM MgSO_4_ and 20 g/L glucose. Error bars represent the range of three independent fermentations.

FPP synthase catalyzes the head-to-tail condensation of IPP with DMAPP as well as with geranyl pyrophosphate (GPP) to yield FPP as the final product^23^. Overexpression of FPP synthase would increase the supply of FPP which has been demonstrated in previous work^24^. In this study, three different FPP synthases (IspA from *E. coli*, IsoA from *Blakeslea trispora* and Erg20 from *Saccharomyces cerevisiae*) were tested to investigate their effects on longifolene production. Due to the difficulty in detecting and quantifying FPP, these FPP synthases were co-expressed with LgfS using the recombinant plasmids pA-ispAlgfS, pA-isoAlgfS or pA-erg20lgfS to directly investigate their effects on longifolene production. The recombinant plasmids were transformed into BL21(DE3) competent cells and cultured in sealed shake flasks. All the three FPP synthases were successfully expressed in *E. coli* (Fig. 2, lane 3 to 5). After being induced with 0.1 mM isopropyl-ß-D-thiogalactopyranoside (IPTG) at 30 °C for 24 h, longifolene production of different strains were shown in Fig. 4. The FPP synthases overexpressing strains produced more longifolene than strain BL21/pA-lgfS. Among them, strain BL21/pA-ispAlgfS produced 1.61 mg/L of longifolene (1.8-fold to strain BL21/pA-lgfS), which was the highest of all. This result demonstrates that the native FPP synthase was the most efficient for FPP supply. *B. trispora* is expected to have an efficient FPP synthase. It is a good natural producer for lycopene and β-carotene used on the industrial scale^25^ and FPP is also the common precursor for the biosynthesis of lycopene and β-carotene. However, only a slight enhancement of longifolene production was observed when *isoA* was co-expressed with *lgfS*. In addition, this fungal FPP synthase seemed not to be correctly expressed or get degraded in *E. coli* since its molecular weight determined by the SDS-PAGE was smaller than its theoretical value. Erg20 of *S. cerevisiae* also encodes an FPP synthase. Overexpression of this FPP synthase has been demonstrated to enhance terpenoids production in yeast strains^26^. However, this enzyme was not as efficient as the native FPP synthase in this prokaryotic system and strain BL21/pA-erg20ispA produced 1.22 mg/L of longifolene, which is much lower than strain BL21/pA-ispAlgfS.

### Enhancement of longifolene production using the MVA pathway

FPP is derived from two universal building blocks, IPP and DMAPP. To further enhance FPP supply and longifolene production, we engineered the recombinant strain to increase the intracellular levels of IPP and DMAPP. *E. coli* utilizes MEP pathway to convert pyruvate and glyceraldehyde-3-phosphate into IPP and DMAPP. Recent studies showed that the MVA pathway was more efficient than MEP pathway for the synthesis of IPP and DMAPP^27^. The MVA pathway could be divided into two parts: the upper pathway (from acetyl-CoA to MVA) and the lower pathway (from MVA to IPP). The upper MVA pathway from *Enterococcus faecalis* has been demonstrated to be very efficient for MVA biosynthesis. Overexpression of the *mvaE* (acetoacetyl-CoA synthase/HMG-CoA reductase) and *mvaS* (HMG-CoA synthase gene) genes of *E. faecalis* led to efficient production of mevalonate in recombinant *E. coli*^28^. We also cloned the *mvaE* and *mvaS* genes into plasmid pACYCDuet-1, generating pA-mvaES. Similar results were obtained by strain BL21/pA-mvaES. Shake-flask cultivation showed that about 2.2 g/L of mevalonate accumulated in the fermentation broth of the engineered strain (Supplementary Figure S2).

Mevalonate is converted to IPP through the lower MVA pathway which consists of mevalonate kinase (MK), pyrophosphomevalonate kinase (PMK) and mevalonate pyrophosphate decarboxylase (MVD) enzymes^29^. The lower MVA pathway from *S. cerevisiae* (encoded by *erg19*, *erg8* and *erg12*) has been well characterized and cloned into the pTrcHis2B plasmid in our previous study, resulting pTrcLower^30^. Biosynthesis of one molecule of longifolene requires two molecules of IPP and one molecule of DMAPP. The ratio of IPP and DMAPP in *E. coli* could not meet this requirement^31^. IPP isomerase (IDI) catalyzes the interconversion between IPP and DMAPP. Overexpression of IDI would result in a proper ratio of IPP and DMAPP, which might contribute to longifolene production. Therefore, the *S. cerevisiae idi1* gene was also cloned into the pTrcLower plasmid. To integrate the longifolene biosynthesis pathway and the MVA pathway, the *ispA* and *lgfS* genes were further cloned into pA-mvaES sequentially, resulting in pA-mvaESispAlgfS. Both of pTrcLower and pA-mvaESispAlgfS were co-transformed into *E. coli* BL21(DE3) and the engineered strain BL21/pTrcLower&pA-mvaESispAlgfS was cultured under the abovementioned conditions to test the ability for longifolene production. The amount of longifolene accumulated of this strain reached 2.64 mg/L after being induced by 0.1 mM IPTG for 24 h, which was 1.6-fold higher than strain BL21/pA-ispAlgfS. The yield of longifolene on glucose reached 0.18% of the theoretical limits (25.2%, 9 Glucose → 2 longifolene). These results indicate that the MVA pathway caused a considerable increase in longifolene production.

### Scale-up of longifolene production by fed-batch fermentation

**Figure 5:**
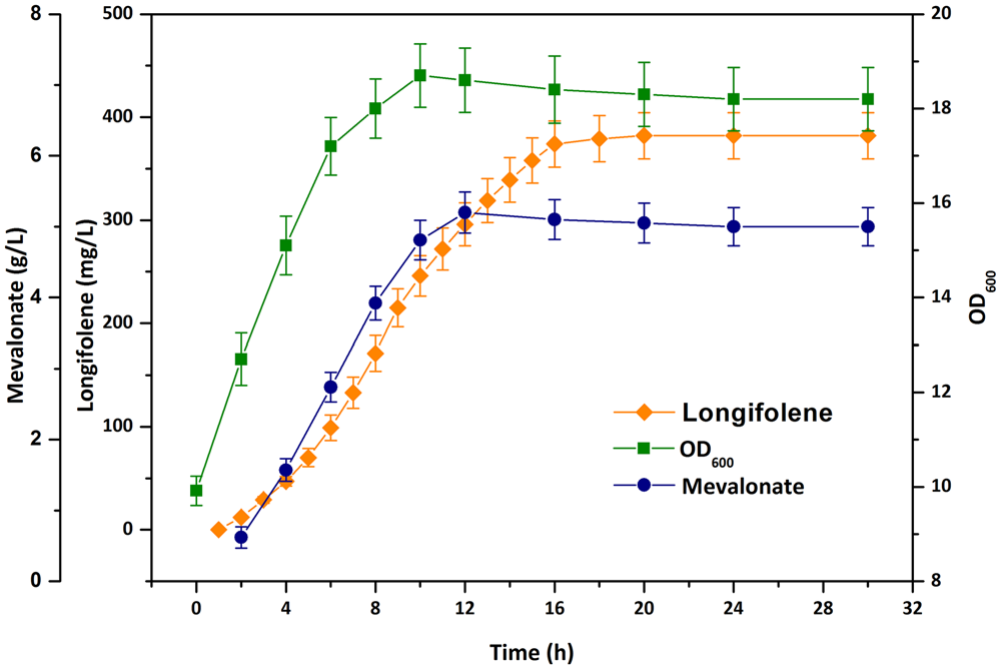
Time-course profiles for cell density (OD_600_), mevalonate and longifolene production during fed-batch fermentation of the best-performing strain BL21/pA-mvaESispAlgfS&pTrcLower. Cultures were performed in a 5 L stirred tank bioreactor. Error bars represent the range of three independent fermentations.

To achieve a high-density cell culture for longifolene production, fed-batch fermentation was carried out using the best-performing strain BL21/pA-mvaESispAlgfS&pTrcLower in a 5 L bioreactor. Based on the concentration of residual glucose, the feeding rate was controlled to maintain it lower than 1 g/L. Figure 5 presents the time-course profiles of cell density, mevalonate and longifolene production during the fermentation processes. Longifolene biosynthesis started from 2 h post-induction and then increased rapidly during the fed-batch process. After 20 h of cultivation, longifolene could be hardly detected in the off-gas. The production of longifolene appeared to be coupled with cell growth and reached a maximum titer of 382 mg/L with a specific productivity of 2.43 mg/(L·h·gDCW) [an OD_600_ of 1.0 corresponds to 0.43 g dry cell weight (DCW) per liter]. However, this engineered strain grew much weaker than normal *E. coli* strains and cell density ceased at an OD_600_ less than 20 even though glucose was still consumed. The poor growth of the engineered strain might be due to accumulation of the toxic intermediates IPP^32,33^ and FPP^34^, which was further confirmed by fed-batch culture of strain BL21/pA-mvaESispA&pTrcLower. In a fermentation process, obtaining higher biomass is a common strategy to increase the product titer. Therefore, the elimination of these toxic intermediates would be helpful to further enhance longifolene production. Longifolene biosynthesis pathway consists of many heterologous genes. Overexpression of these genes in multiple plasmids was a huge metabolic burden on the host cells, which might also decrease the cell growth rate^35^. Stable and long-term longifolene producing system may be accomplished by integrating these genes into the chromosome of the *E. coli* host^36^. In addition, the intermediate metabolite mevalonate also gradually accumulated in the culture broth and reached a maximum titer of 5.2 g/L. Most of the mevalonate cannot be efficiently converted to longifolene. This result suggests that the upper pathway produced too much mevalonate beyond the capacity of the lower pathway. The metabolic unbalance between the upper and lower pathway also contributed to the poor cell growth.

## Discussion

Naturally occurring terpenes are getting considerable research attentions in recent years. Sesquiterpenes represent a class of highly reactive volatile terpenes that consist of three isoprene units^37,38^. As a typical tricyclic sesquiterpene, the expanding applications and growing demands of longifolene have led to an extensive search for efficient and economical manufacturing methods. Microorganisms have recently been recognized as potentially important sources of terpenes and can provide a reliable way for large-scale production^39^. There is growing interest in engineering terpene production strains to overcome the limited supply of these commercially valuable compounds. In this study, we used the model organism *E. coli* as the host and preliminarily proved the feasibility of microbial longifolene production. However, the current titer and productivity of longifolene is still too low for industrial applications.

The last reaction catalyzed by LgfS should be the rate-limiting step during longifolene biosynthesis. The activity of this enzyme was much lower than many other terpene synthases due to the complex carbocation polycyclization reaction to form the tricyclic structure^40^. Although the LgfS was codon optimized to enhance its expression levels, this strategy was not enough to achieve a finally industrial level production. Up to now, the structure of LgfS has not been resolved. But recent advancements in understanding the reaction mechanism of terpene synthases can guide developing mutant LgfS enzymes with improved activity. Screening for more efficient LgfS^41^ or using protein engineering strategy^42^ to improve its catalytic activity, which is currently being undertaken in our lab, have the potential to enhance the productivity of this valuable sesquiterpene.

The low intracellular IPP and DMAPP availability also restricts the efficient production of terpenoids in *E. coli*^43^. One of the key strategies to construct an efficient host for terpenoids biosynthesis is to introduce a heterologous MVA pathway. Here, we expressed a hybrid MVA pathway in *E. coli* which has been demonstrated to be effective for isoprene production in our previous study^44^. Due to the augumentation of IPP and DMAPP pools, longifolene production was enhanced to some extent. Although not pursued in this study, optimal expression levels of the MVA pathway enzymes will be beneficial for further increasing longifolene production. IPP and DMAPP are not the direct substrates for longifolene biosynthesis. They are condensed to form the C15 unit of FPP, which is the precursor of sesquiterpenes. FPP synthases, catalyzing the formation of both GPP and FPP, is a key enzyme in the sesquiterpenes biosynthesis pathway^45^. After comparison of three different FPP synthases, we established a whole pathway for the precursor biosynthesis from simple building blocks. Longifolene production was further improved by fed-batch fermentation of the engineered strain.

Energy and redox balance is critical for microorganisms, but often disrupted by the introduction of exogenous synthetic pathways. The reducing equivalent demand and ATP requirement play an important role in longifolene biosynthesis. Longifolene could be produced from either the native *E. coli* MEP pathway or the exogenous MVA pathway. MEP pathway has a higher theoretical yield but requires ATP and NADPH for metabolic balance^46^. MVA pathway is more energetically favorable. The dissipation of NADPH and ATP in the MVA pathway could be covered by the upper glycolysis pathway^47^. A net of 12 molecules of NADH can be generated when one molecule of longifolene is synthesized (Fig. 1). Therefore, the coupling of the complementary reducing equivalent demand and ATP requirement in the synergy of MEP and MVA pathway would greatly influence longifolene biosynthesis^48^. Future work balancing the reducing equivalent and ATP of the two pathways has the potential to further improve the productivity of longifolene.

In conclusion, longifolene production was achieved by assembling biosynthetic genes encoding a heterologous MVA pathway, a native FPP synthase and a longifolene synthase in an engineered *E. coli* strain. The best-performing strain BL21/pA-mvaESispAlgfS&pTrcLower accumulated up to 382 mg/L of longifolene under fed-batch fermentation conditions. An alternative producing system for longifolene from renewable resources via microbial fermentation has been provided. This study can serve as the basis for the construction of much more robust strains for sesquiterpene production in the future.

## Methods

### Strains, media and culture conditions

A list of bacterial and yeast strains and recombinant plasmids used in this study is presented in Table 1. *E. coli* DH5α was grown in LB medium (10 g/L tryptone, 5 g/L yeast extract and 5 g/L NaCl) for plasmids construction. *E. coli* BL21(DE3) was grown in M9 mineral medium (6 g/L Na_2_HPO_4_, 3 g/L KH_2_PO_4_, 1 g/L NH_4_Cl and 0.5 g/L NaCl) supplemented with 1 mM MgSO_4_ and 20 g/L glucose as the carbon source for longifolene production. Growth medium (9.8 g/L K_2_HPO_4_·3H_2_O, 3 g/L (NH_4_)_2_SO_4_, 2.1 g/L citric acid monohydrate and 0.3 g/L ferric ammonium citrate) supplemented with 20 g/L glucose, 1 mM MgSO_4_ and 1 ml/L trace elements (3.7 g/L (NH_4_)_6_Mo_7_O_24_·4H_2_O, 2.9 g/L ZnSO_4_·7H_2_O, 24.7 g/L H_3_BO_3_, 2.5 g/L CuSO_4_·5H_2_O, 15.8 g/L MnCl_2_·4H_2_O) was used for fed-batch fermentation. Appropriate antibiotics were added to the culture medium if necessary at the following concentrations: 100 μg/ml ampicillin or 34 μg/ml chloramphenicol or both of them.

**Table 1:** Strains and plasmids used in this study.

Strains or plasmids	Genotype/Description	Sources
**Strains**		
*E. coli* DH5α	*F* ^−^ *recA endA1 Φ80dlacZΔM15 hsdR17(r*_*K*_^−^ *m*_*K*_^+^ *) λ* ^−^	Invitrogen
*E. coli* BL21(DE3)	*F*^−^ *ompT hsdS*_*B*_ (r_B_^−^ m_B_^−^) *gal dcm rne131* (DE3)	Invitrogen
*S. cerevisiae 288c*	*MATα SUC2 gal2 mal2 mel flo1 flo8-1 hap1 ho bio1 bio6*	American type culture collection (ATCC)
*B. trispora* DSM-2388	*Mating type -*	German Collection of Microorganisms and Cell Cultures (DSMZ)
**Plasmids**		
pACYCDuet-1	*Cm* ^*r*^ *oriP15A lacI* ^*q*^ *T7p*	Novagen
pET30a(+)	*Kan* ^*r*^ *oripBR322 lacI* ^*q*^ *T7p*	Novagen
pEASY-Blunt	*Kan* ^*r*^ *Amp* ^*r*^ *oripUC*	Transgen
pTrcHis2B	*Amp* ^*r*^ *ori pBR322 lacI* ^*q*^ *Trcp*	Invitrogen
pUC-lgfS	pUC57 harboring codon optimized *P. abies lgfS*	This study
pET-lgfS	pET30a harboring *P. abies lgfS*	This study
pA-lgfS	pACYCDuet-1 harboring *P. abies lgfS*	This study
pEasy-ispA	pEASY-Blunt harboring *E. coli ispA*	This study
pEasy-isoA	pEASY-Blunt harboring *B. trispora isoA*	This study
pEasy-erg20	pEASY-Blunt harboring *S. cerevisiae erg20*	This study
pA-ispAlgfS	pACYCDuet-1 harboring *E. coli ispA* and *P. abies lgfS*	This study
pA-isoAlgfS	pACYCDuet-1 harboring *B. trispora isoA* and *P. abies lgfS*	This study
pA-erg20AlgfS	pACYCDuet-1 harboring *S. cerevisiae erg20* and *P. abies lgfS*	This study
pTrcLower	pTrcHis2B harboring *S. cerevisiae erg19*, *erg8*, *erg12* and *idi1*	^30^
pA-mvaES	pACYCDuet-1 harboring *E. faecalis mvaE* and *mvaS*	^30^
pA-mvaESispA	pACYCDuet-1 harboring *E. faecalis mvaE*, *mvaS* and *E. coli ispA*	This study
pA-mvaESispAlgfS	pACYCDuet-1 harboring *E. faecalis mvaE*, *mvaS*, *E. coli ispA* and *P. abies lgfS*	This study

### Plasmids construction

The primers used for plasmids construction are given in Table 2. The gene encoding longifolene synthase (*lgfS*, GenBank Accession No.: AY473625) from *P. abies* was codon optimized using an online tool (www.jcat.de), chemically synthesized and cloned into pUC57 vector by Genewiz Biotech Co., Ltd. (Nanjing, China). Then *lgfS* was PCR amplified and subcloned into the restriction sites *Nde*I/*Bgl*II of vector pET30a(+) or pACYCDuet-1, creating pET-lgfS or pA-lgfS. Three different FPP synthases were investigated to enhance longifolene production. The *ispA* gene of *E. coli* and *erg20* gene of *S. cerevisiae* were amplified using genomic DNA as the template and directly ligated into the pEASY-Blunt vector (Transgen, China), resulting pEasy-ispA and pEasy-erg20. The *isoA* gene (GenBank Accession No.: JQ289994) of *B. trispora* was amplified using total RNA as the template by a RT-PCR Kit (TaKaRa, China) and also cloned into the pEASY-Blunt vector, resulting pEasy-isoA. Then these FPP synthases were subcloned into pA-lgfS to create pA-ispAlgfS, pA-isoAlgfS and pA-erg20lgfS. Two recombinant plasmids, pA-mvaES harboring *E. faecalis mvaE* and *mvaS* (encoding the MVA upper pathway), and pTrcLower harboring *S. cerevisiae erg19*, *erg8, erg12* and *idi1* (encoding the MVA lower pathway) were constructed in our previous work^30^. The *ispA* gene was further inserted into the corresponding sites *Bgl*II/*Pvu*I of the vector pA-mvaES to create pA-mvaESispA. Finally, PCR reaction was performed using pET-lgfS as the template and a primer pair that allowed the amplification of the T7 promoter along with the *lgfS* structure gene. The PCR product, T7lgfS was then cloned into pA-mvaESispA between *Pvu*I and *Xho*I sites, to create pA-mvaESispAlgfS. All the expression constructs were illustrated in Supplementary Figure S3.

**Table 2:** Primers used in this study for plasmids construction.

Oligonucleotide primers	Sequences
lgfS_F_NdeI	GGGAATTCCATATGGCTCAGATCAGTAAATGCTC
lgfS_R_BglII	GGAAGATCTTAGGTCAGCGGGTCGATCAG
ispA_F_NcoI	CCATGGACTTTCCGCAGCAACTC
ispA_R_BamHI	GGATCCTTATTTATTACGCTGGATGATGTAGTCC
isoA_F_NcoI	CCATGGTTGCTGTCAAGTTACCAAAG
isoA _R_SalI	GTCGAC TTATTTAGTACGCTTGTAGATCTTG
erg20_F_NcoI	CCATGGCTTCAGAAAAAGAAATTAGGAG
erg20_R_BamHI	GGATCCCTATTTGCTTCTCTTGTAAACTTTGTTC
ispA_F_BglII	GGAAGATCTCATGGACTTTCCGCAGCAACTC
ispA_R_PvuI	TCGCGATCGTTATTTATTACGCTGGATGATGTAGTCC
T7lgfS_F_PvuI	TCGCGATCGTAATACGACTCACTATAGGGGAATTGTG
T7lgfS_R_XhoI	CCGCTCGAGTTAGGTCAGCGGGTCGATCAGGATTTTG

The restriction sites in the primers were underlined.

### Protein expression and gel electrophoresis analysis

*E. coli* BL21(DE3) harboring different recombinant plasmids was grown on LB agar plates. A single colony was used to inoculate 5 ml of liquid LB medium, which was incubated overnight at 37 °C and 180 rpm. The saturated culture was diluted 1:100 in fresh LB medium and incubated under the same conditions. When the optical density at 600 nm (OD_600_) reached about 0.6, IPTG was added to a final concentration of 0.1 mM, and cell growth was continued for 4 h. Bacterial cell pellets obtained from 3 ml of culture were suspended in 0.1 mM phosphate-buffered saline (PBS) (pH 7.2) and disrupted with a high-intensity ultrasonic processor (VCX-130PB, Sonics & Materials). The mixture was centrifuged and the supernatant obtained was mixed with 2 × loading buffer, heated at 100 °C for 5 min and then analyzed by SDS- PAGE.

### Shake-flask fermentation

Shake-flask cultures were performed in 600 ml sealed bottles containing 50 ml liquid M9 mineral medium. *E. coli* strains harboring different recombinant plasmids were inoculated to the culture medium and incubated in a gyratory shaker incubator at 37 °C and 180 rpm. 0.1 mM of IPTG was added to induce recombinant proteins expression at an OD_600_ reached about 0.6. Then the temperature was shifted to 30 °C for longifolene production. As terpenes are volatile compounds and toxic to bacterial cells, two-phase fermentation^49^ was further employed to extract longifolene from the aqueous culture broth and reduce volatilization. For two-phase fermentation, 10% (v/v) of decane was added to the culture broth after IPTG induction as an overlay. The culture was further incubated for 24 h. Then, samples were taken to determine cell density, residual glucose, mevalonate and longifolene production.

### Fed-batch fermentation

For large-scale production of longifolene, fed-batch cultivation was carried out in a 5 L working volume stirred tank bioreactor (Biostat B plus MO5L, Sartorius, Germany) containing 3 L of growth medium. 50 ml of seed culture was prepared by incubating in shake flasks overnight at 37 °C. Sparger aeration was employed using filtered air to maintain the dissolved oxygen (DO) concentration. During the fermentation process, the pH was controlled at 7.0 via automated addition of ammonia. The fermentation was first operated in a batch mode and the control settings were: 37 °C, agitation rate at 200 rpm and airflow at 1 vvm. Antifoam (polyoxyethylene polyoxypropylene polyol ethers) was automatically added in case of foam development during the fermentation courses. The agitation was associated with DO to maintain a DO concentration above 20% saturation. When the cells were grown to an OD_600_ of about 10, the culture temperature was switched to 30 °C and IPTG was added to the culture broth at a final concentration of 0.1 mM. After the initial glucose was exhausted which was indicated by the sharp rise of DO, fed-batch mode was commenced by feeding a solution containing 65% of glucose at appropriate rates and the residual glucose was maintained at a low level to control the formation of acetic acid, the most deleterious by-product for *E. coli* fermentation. Samples of fermentation broth were withdrawn at appropriate intervals to determine cell density, residual glucose and mevalonate production. Longifolene accumulation was measured every 60 min by analyzing the off-gas using GC.

### Analytic methods

Cell growth of the *E. coli* culture in shake-flasks or bioreactors was determined by measuring OD_600_ of appropriate dilutions using a spectrophotometer (Cary 50 UV-vis, Varian).

The residual glucose in the culture broth was quantified using a Biological Sensing Analyzer (SBA-40D, Biology Institute of Shandong Academy of Sciences, China).

Longifolene was identified using an Agilent GC-MS system (7890 A/5975 C). The GC-MS conditions were as follows: a 30 m HP-INNOWAX column (internal diameter 0.25 mm, film thickness 0.25 μm); an oven temperature program composed of an initial hold at 100 °C for 2 min, ramping at 10 °C/min to 250 °C, and a final hold at 250 °C for 5 min; high-purity helium as the carrier gas with a linear velocity of 1 ml/min; an injector temperature of 250 °C; a split ratio of 1:20; an ion source temperature of 230 °C, EI ionization at 70 eV and mass range of m/z 35–300. The peak of longifolene was identified by the retention time of an external standard and mass spectral comparison with a National Institute of Standards and Technology (NIST) database.

The contents of longifolene and the metabolic intermediate mevalonate were measured using an Agilent 7890B GC system equipped with a flame ionization detector (FID) and a 30 m HP-5 column (internal diameter 0.32 mm, film thickness 0.25 μm) and using high-purity nitrogen as the carrier gas with a flow rate of 1 ml/min. The injector temperature was 250 °C, the FID temperature was 250 °C and the split ratio was 1:10. The oven was initially set at 100 °C for 1 min, increased at 20 °C/min to 160 °C, then 10 °C/min to 250 °C and finally held at 250 °C for 3 min. For mevalonate determination, fresh culture broth was centrifuged at 12,000 g for 10 min, and the supernatants were adjusted to a pH below 2 and incubated at 45 °C for 1 h to convert mevalonate to its lactone form. Then the mixture was saturated with anhydrous Na_2_SO_4_ and mevalonate lactone was extracted with ethyl acetate^50^. The resulting organic layers were analyzed by GC to determine mevalonate production. For longifolene determination, the analytic methods were adjusted according to the fermentation strategy. For normal fermentation, two different methods were used to determine longifolene concentration: extracting the products in the fermentation broth or analyzing the gas in the sealed shake flasks. To extract longifolene in the fermentation broth, 10% (v/v) of decane was added to the culture broth at the end of fermentation. Then the decane phase of was collected and centrifuged for to remove celldebris and subsequently subjected to GC. To determine longifolene production in the gas phase, the sealed cultures were kept in a 37 °C water bath for 5 min after 24 h fermentation. Then 1 ml of gas sample was taken from the headspace of the sealed cultures and analyzed using GC. For two-phase fermentation, the decane phase was directly collected and analyzed using GC.

## Electronic supplementary material

Supplementary data
