# Supplementary material for: Manipulation of the precursor supply for high-level production of longifolene by metabolically engineered Escherichia coli
Source: Sci Rep. 2019 Jan 14;9:95. doi: 10.1038/s41598-018-36495-w (PMC6331559; doi:10.1038/s41598-018-36495-w)
Supplement: Supplementary file 1 — Supplementary data [file 41598_2018_36495_MOESM1_ESM.pdf]

Manipulation of the precursor supply for high-level production of longifolene by metabolically engineered *Escherichia coli*

Yujin Cao [1,*], Rubing Zhang [1,*], Wei Liu [1], Guang Zhao [1], Wei Niu [2], Jiantao Guo [2], Mo Xian [1], Huizhou Liu [1]

[1] CAS Key Laboratory of Biobased Materials, Qingdao Institute of Bioenergy and Bioprocess Technology, Chinese Academy of Sciences, Qingdao, China

[2] Department of Chemistry, University of Nebraska-Lincoln, Lincoln, Nebraska, United States

Supplementary figure captions

Figure S1 Codon optimization of the longifolene synthase gene. wlgfs, wild-type longifolene synthase gene; olgfs, codon-optimized longifolene synthase gene. The identical codons are shown in dark blue and the optimized codons are shown in light blue. The amino acid encoded by each codon is shown as its abbreviation.

Figure S2 Time-course profiles for cell density (OD_{600}) and mevalonate production during shake-flasks culture of strain BL21/pA-mvaES. Cultures were performed in 500 ml shake flasks containing 50 ml liquid M9 mineral medium supplemented with 1 mM $MgSO_4$ and 20 g/L glucose. Error bars represent the range of three independent fermentations.

Figure S3 Schematic diagram of the expression constructs containing the genes encoding longifolene biosynthesis pathway. lgfS, longifolene synthase; ispA, *E. coli* FPP synthase; isoA, *B. trispora* FPP synthase; erg20, *S. cerevisiae* FPP synthase; mvaE, acetoacetyl-CoA synthase/HMG-CoA reductase; mvaS, HMG-CoA synthase gene; erg19, mevalonate kinase; erg8, phosphomevalonate kinase; erg12, mevalonate pyrophosphate decarboxylase; idi1, IPP isomerase.

Figure S1

```
wlgfS   ATGGCTCAGATTTCTAAATGTTCGTCACTGTCCGCCGAGTTGAACGAATCCTCCATCATC   60
         M  A  Q  I  S  K  C  S  S  L  S  A  E  L  N  E  S  S  I  I
olgfS   ATGGCTCAGATCAGTAAATGCTCTTCTCTGTCTGCTGAACTGAACGAATCTTCTATCATC   60

wlgfS   TCTCATCATCATGGAAATCTGTGGGACGACGATTTCATACAATCTCTTAAGTCGTCCAAT   120
         S  H  H  H  G  N  L  W  D  D  D  F  I  Q  S  L  K  S  S  N
olgfS   TCTCACCACCACGGTAACCTGTGGGACGACGACTTCATCCAGTCTCTGAAATCTTCTAAC   120

wlgfS   GGGGCACCTCAATATCATGAACGCGCTGCGAAACTTGTTGAAGAAATCAAGAATTTAGTT   180
         G  A  P  Q  Y  H  E  R  A  A  K  L  V  E  E  I  K  N  L  V
olgfS   GGTGCTCCGCAGTACCACGAACGTGCTGCTAAACTGGTTGAAGAAATCAAAAACCTGGTT   180

wlgfS   GTGAGCGAGATGAAAGATTGCAACGATGATTTAATCAGACGTCTTCAGATGGTTGACATT   240
         V  S  E  M  K  D  C  N  D  D  L  I  R  R  L  Q  M  V  D  I
olgfS   GTTTCTGAAATGAAAGACTGCAACGACGACCTGATCCGTCGTCTGCAGATGGTTGACATC   240

wlgfS   TTTGAATGTCTAGGAATTGATCGGCACTTTCAGCATGAAATACAAGTTGCTCTTGATTAC   300
         F  E  C  L  G  I  D  R  H  F  Q  H  E  I  Q  V  A  L  D  Y
olgfS   TTCGAATGCCTGGGTATCGACCGTCACTTCCAGCACGAAATCCAGGTTGCTCTCGACTAC   300

wlgfS   GTGTACAGATATTGGAACCAGCTGGAAGGCATCGGTATTGGATCAAGAGATTCCCTTATC   360
         V  Y  R  Y  W  N  Q  L  E  G  I  G  I  G  S  R  D  S  L  I
olgfS   GTCTACCGCTACTGGAACCAGCTGGAAGGTATCGGTATCGGTTCTCGTGACTCTCTGATC   360

wlgfS   AAAGATTTCAATGCTACAGCTTTGGGATTTCGGGCTCTCCGACTGCATCGATATAACGTT   420
         K  D  F  N  A  T  A  L  G  F  R  A  L  R  L  H  R  Y  N  V
olgfS   AAAGACTTCAACGCTACCGCTCTGGGTTTCCGTGCTCTGCGTCTGCACCGTTACAACGTT   420

wlgfS   TCCTCAGATGTCTTGGAGAATTTCAAGAACGAAAACGGGCAGTTCTTCTGCAGTTCCACG   480
         S  S  D  V  L  E  N  F  K  N  E  N  G  Q  F  F  C  S  S  T
olgfS   TCTTCTGACGTTCTGGAAAACTTCAAAAACGAAAACGGTCAGTTCTTCTGCTCTTCTACC   480

wlgfS   GTTGAAGAAAAAGAAGTGAGATGCATGTTGACGTTATTTCGAGCTTCAGAAATTTCATTT   540
         V  E  E  K  E  V  R  C  M  L  T  L  F  R  A  S  E  I  S  F
olgfS   GTTGAAGAAAAAGAAGTTCGTTGCATGCTGACCCTGTTCCGTGCTTCTGAAATCTCTTTC   540

wlgfS   CCCGGAGAAAAAGTGATGGACGAGGCAAAGGCGTTCACAACAGAATATCTAACCAAAGTT   600
         P  G  E  K  V  M  D  E  A  K  A  F  T  T  E  Y  L  T  K  V
olgfS   CCGGGTGAAAAAGTTATGGACGAAGCTAAAGCTTTCACCACCGAATACCTGACCAAAGTT   600

wlgfS   TTAACGGGAGTGGATGTAACGGACGTCAATCAAAGCCTTTTGAGAGAAGTGAAGTATGCC   660
         L  T  G  V  D  V  T  D  V  N  Q  S  L  L  R  E  V  K  Y  A
olgfS   CTGACCGGTGTTGACGTTACCGACGTTAACCAGTCTCTGCTGCGTGAAGTTAAATACGCT   660

wlgfS   CTGGAGTTTCCATGGCATTGCAGTTTGCCGAGATGGGAGGCAAGGAGCTTTATCGAAATA   720
         L  E  F  P  W  H  C  S  L  P  R  W  E  A  R  S  F  I  E  I
olgfS   CTGGAATTCCCGTGGCACTGCTCTCTGCCGCGTTGGGAAGCTCGTTCTTTCATCGAAATC   720

wlgfS   TGTGGACAAAACGATTCATGGCTCAAGTCGATTATGAACAAACGAGTTTTAGAGTTGGCG   780
         C  G  Q  N  D  S  W  L  K  S  I  M  N  K  R  V  L  E  L  A
olgfS   TGCGGTCAGAACGACTCTTGGCTGAAATCTATCATGAACAAACGTGTTCTGGAACTGGCT   780

wlgfS   AAATTGGACTTCAATATTTTGCAATGGGCACATCATAGAGAACTACAGCTTCTGTCAAGT   840
         K  L  D  F  N  I  L  Q  W  A  H  H  R  E  L  Q  L  L  S  S
olgfS   AAACTGGACTTCAACATCCTGCAGTGGGCTCACCACCGTGAACTGCAGCTGCTGTCTTCT   840

wlgfS   TGGTGGTCACAATCGGATATAGCGCAGCAGAATTTCTATCGGAAGCGTCACGTGGAATTT   900
         W  W  S  Q  S  D  I  A  Q  Q  N  F  Y  R  K  R  H  V  E  F
olgfS   TGGTGGTCTCAGTCTGACATCGCTCAGCAGAACTTCTACCGTAAACGTCACGTTGAATTC   900

wlgfS   TACTTATGGGTGGTTATAGGCACGTTCGAGCCGGAGTTTTCAACATGCAGAATCACCTTT   960
         Y  L  W  V  V  I  G  T  F  E  P  E  F  S  T  C  R  I  T  F
olgfS   TACCTGTGGGTTGTTATCGGTACCTTCGAACCGGAATTCTCTACCTGCCGTATCACCTTC   960

wlgfS   GCAAAAATCTCTACACTGATGACTATTCTAGATGACCTCTACGATACTCACGGAACGTTG   1020
         A  K  I  S  T  L  M  T  I  L  D  D  L  Y  D  T  H  G  T  L
olgfS   GCTAAAATCTCTACCCTGATGACCATCCTGGACGACCTGTACGACACCCACGGTACCCTG   1020

wlgfS   GAACAACTCAAAATCTTCACAGAGGGAGTGAAACGATGGGATCTTTCGTTAGTAGACCGC   1080
         E  Q  L  K  I  F  T  E  G  V  K  R  W  D  L  S  L  V  D  R
olgfS   GAACAGCTGAAAATCTTCACCGAAGGTGTTAAACGTTGGGACCTGTCTCTGGTTGACCGT   1080

wlgfS   CTTCCAGACTACATAAAGATTACATTCGAATTCTTCCTCAACACATCTAATGAATTGATT   1140
         L  P  D  Y  I  K  I  T  F  E  F  F  L  N  T  S  N  E  L  I
olgfS   CTGCCGGACTACATCAAAATCACCTTCGAATTCTTCCTGAACACCTCTAACGAACTGATC   1140

wlgfS   GCTGAAGTTGCGAAAACGCAAGAGCGGGATATGTCAGCCTACATACGAAAAACATGGGAA   1200
         A  E  V  A  K  T  Q  E  R  D  M  S  A  Y  I  R  K  T  W  E
olgfS   GCTGAAGTTGCTAAAACCCAGGAACGTGACATGTCTGCTTACATCCGTAAAACCTGGGAA   1200

wlgfS   CGATACCTTGAAGCTTATTTGCAAGAAGCGGAATGGATAGCAGCTCGACACGTCCCTACG   1260
         R  Y  L  E  A  Y  L  Q  E  A  E  W  I  A  A  R  H  V  P  T
olgfS   CGTTACCTGGAAGCTTACCTGCAGGAAGCTGAATGGATCGCTGCTCGTCACGTTCCGACC   1260

wlgfS   TTTGATGAGTACATGAAGAATGGCATATCCAGTTCTGGGATGTGTATACTAAATTTGTAT   1320
         F  D  E  Y  M  K  N  G  I  S  S  S  G  M  C  I  L  N  L  Y
olgfS   TTCGACGAATACATGAAAAACGGTATCTCTTCTTCTGGTATGTGCATCCTGAACCTGTAC   1320

wlgfS   TCGCTTCTGTTAATGGGGCAACTTCTACCTGACGACGTTCTGGAGCAAATACACTCTCCA   1380
         S  L  L  L  M  G  Q  L  L  P  D  D  V  L  E  Q  I  H  S  P
olgfS   TCTCTGCTGCTGATGGGTCAGCTGCTGCCGGACGACGTTCTGGAACAGATCCACTCTCCG   1380

wlgfS   TCCAAGATCCACGAGCTTGTAGAATTGACGGCCAGACTGGTAGACGACTCAAAGGATTTT   1440
         S  K  I  H  E  L  V  E  L  T  A  R  L  V  D  D  S  K  D  F
olgfS   TCTAAAATCCACGAACTGGTTGAACTGACCGCTCGTCTGGTTGACGACTCTAAAGACTTC   1440

wlgfS   GAGACGAAGAAGGTTGGTGGGGAGTTAGCTTCAGGTATAGAGTGTTACGTGAAAGACAAC   1500
         E  T  K  K  V  G  G  E  L  A  S  G  I  E  C  Y  V  K  D  N
olgfS   GAAACCAAAAAAGTTGGTGGTGAACTGGCTTCTGGTATCGAATGCTACGTTAAAGACAAC   1500

wlgfS   CCTGAATGTACACTGGAAGATGCTTCAAATCATCTAAATGGCCTCCTTGATCTTACCGTT   1560
         P  E  C  T  L  E  D  A  S  N  H  L  N  G  L  L  D  L  T  V
olgfS   CCGGAATGCACCCTGGAAGACGCTTCTAACCACCTGAACGGTCTGCTGGACCTGACCGTT   1560

wlgfS   AAGGAATTGAATTGGGAGTTTGTAAGGCATGACAGCGTGGCCTTGTGTTTCAAGAAGTTC   1620
         K  E  L  N  W  E  F  V  R  H  D  S  V  A  L  C  F  K  K  F
olgfS   AAAGAACTGAACTGGGAATTCGTTCGTCACGACTCTGTTGCTCTGTGCTTCAAAAAATTC   1620

wlgfS   GCCTTCAACGTTGCACGAGGCCTCCGACTTATCTACAAATACAGAGACGGCTTTGACGTT   1680
         A  F  N  V  A  R  G  L  R  L  I  Y  K  Y  R  D  G  F  D  V
olgfS   GCTTTCAACGTTGCTCGTGGTCTGCGTCTGATCTACAAATACCGTGACGGTTTCGACGTT   1680

wlgfS   TCTAACCAGGAGATGAAAACCCACATATTCAAAATTCTCATCGATCCACTTACTTAG      1737
         S  N  Q  E  M  K  T  H  I  F  K  I  L  I  D  P  L  T  *
olgfS   TCTAACCAGGAAATGAAAACCCACATCTTCAAAATCCTGATCGACCCGCTGACCTAA      1737
```

Figure S2

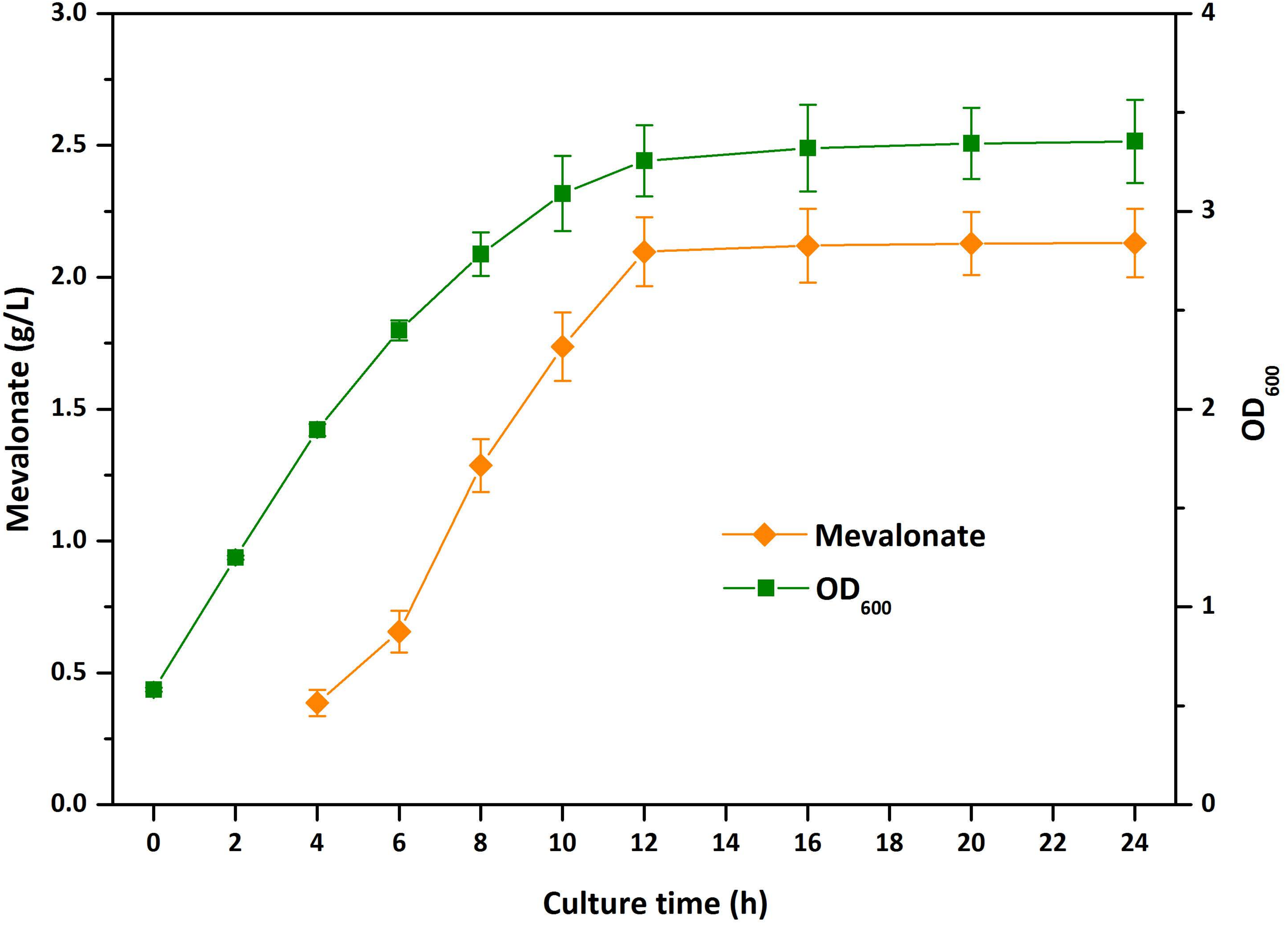

Mevalonate (g/L)
3.0
2.5
2.0
1.5
1.0
0.5
0.0
OD600
4
3
2
1
0
0
2
4
6
8
10
12
14
16
18
20
22
24
Culture time (h)
Mevalonate
OD600

Figure S3

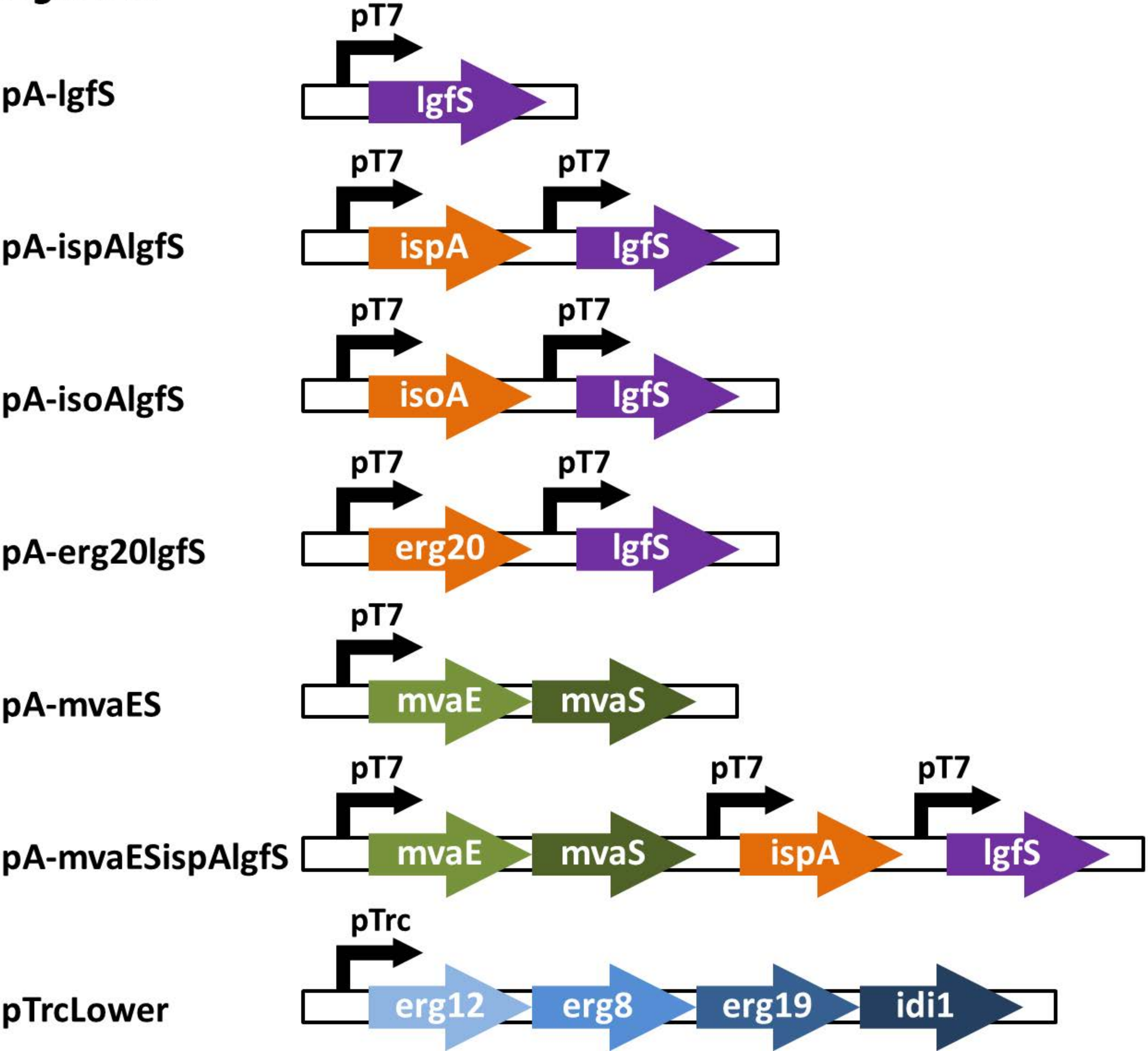

pA-lgfS
pT7
lgfS
pA-ispAlgfS
pT7
ispA
pT7
lgfS
pA-isoAlgfS
pT7
isoA
pT7
lgfS
pA-erg20lgfS
pT7
erg20
pT7
lgfS
pA-mvaES
pT7
mvaE
mvaS
pA-mvaESispAlgfS
pT7
mvaE
mvaS
pT7
ispA
pT7
lgfS
pTrcLower
pTrc
erg12
erg8
erg19
idi1
